# Influence of the Occipital Orientation on Cervical Sagittal Alignment: A Prospective Radiographic Study on 335 Normal Subjects

**DOI:** 10.1038/s41598-018-33287-0

**Published:** 2018-10-18

**Authors:** Weiguo Zhu, Shifu Sha, Zhen Liu, Yang Li, Leilei Xu, Wen Zhang, Yong Qiu, Zezhang Zhu

**Affiliations:** Department of Spine Surgery, Drum Tower Hospital, Medical School of Nanjing University, Nanjing, China

## Abstract

Cervical sagittal alignment is considered to have a special role in the pathogenesis and evaluation of cervical diseases. Previous studies have demonstrated that cervical sagittal alignment is correlated with thoracolumbar and pelvic alignments. As the direct anatomical structures connect to the cervical region, however, no parameters in the occiput have been reported to be intimately related to the alignment of the cervical spine. A retrospective radiographic study of 335 individuals (182 males and 153 females) between 18 and 60 years of age was performed between January 2007 and January 2016. The occipital incidence (OI), occipital slope (OS), occipital tilt (OT), C0-C2 angle, C2-C7 angle, C0-C7 angle, T1 slope and thoracic kyphosis were measured in every individual. The mean values of the occipital parameters of the cohort were 34.6° ± 3.1° for OI, 23.4° ± 7.4° for OS, and 11.8° ± 3.3° for OT. OI was constant throughout three age groups. No significant differences were noted between males and females. In addition, strong correlations were found between the occipital parameters and cervical parameters. The occipital orientation was an important factor that influenced the cervical sagittal alignment.

## Introduction

Over the past a few decades, increasing attention has been paid to the role of cervical sagittal alignment in the pathogenesis and evaluation of cervical diseases. Previous findings have suggested that cervical spine curvature is associated with neck pain, cervical spondylosis and degenerative spondylolisthesis^1–4^. In a series of 124 cases, Massicotte *et al*.^5^ reported the relationship between the preoperative cervical sagittal profile and severity of cervical spinal cord abnormalities in magnetic resonance imaging. Wu *et al*.^6^ found that an improvement in cervical lordosis exerted a significant impact on the long-term clinical outcome after anterior cervical fusion. Therefore, it is important to make a clear assessment of the cervical sagittal alignment, which can facilitate a better understanding of cervical disorders.

To date, the factors that influence the cervical sagittal alignment have not been clearly defined. Hardacker *et al*.^7^ reported a strong correlation between the thoracic sagittal curve and cervical sagittal alignment (C2-C7 angle) in 100 adult volunteers who had cervical lordosis (C2-C7 angle) that increased as thoracic kyphosis (TK) increased. Lee *et al*.’s^8,9^ study further found that the T1 slope (T1S) was the direct factor that affected the cervical sagittal balance (C2-C7 angle) in 77 asymptomatic adults. However, few studies have focused on the relationship between the occipital orientation and cervical spine alignment. Recently, Kim *et al*.^10^ put forward the novel occipital parameter-occipital incidence (OI), which is considered to be an anatomical morphometric parameter like pelvic incidence (PI)^11^ and is associated with the cervical sagittal alignment. However, there was no geometrical equality relationship in the occipital orientation as in the pelvic parameters according to their definition. In addition, the correlation between the occipital orientation and cervical sagittal alignment has not been revealed until now.

In this study, we redefined the occipital parameters to make OI the algebraic sum of the occipital slope (OS) and occipital tilt (OT) and attempted to explore the features of occipitocervical alignment to analyse the correlations among occipitocervical parameters and to provide reference data to further study the cervical sagittal alignment in a large cohort.

## Materials and Methods

### Subjects

This study was approved by the ethics review board of the affiliated Drum Tower Hospital of Nanjing University, and all of the methods were performed in accordance with the guidelines and regulations of the ethics review board. A retrospective radiographic analysis of the occipital orientation and cervical sagittal alignment was performed at our centre between January 2007 and January 2016. Five-hundred-seventy-five subjects in the PACS database with standing neutral lateral cervical radiographs and standing neutral lateral radiographs of the whole spine were included. Radiographs were obtained in a natural standing upright position with the head and trunk vertical and looking straight ahead. Raising (looking up) or lowering (looking down) one’s head was avoided. The inclusion criteria of the subjects were as follows: (1) age between 18 and 60 years and (2) a horizontal gaze. If the Frankfart plane^12^ (an extended line connecting the lower border of the orbit and the external auditory meatus) was horizontal, then the gaze was considered to be horizontal. The exclusion criteria were as follows: (1) any abnormal changes in the occiput or spine in the radiographs; (2) cervical hyperextension or hyperflexion in the cervical radiographs; (3) a kyphotic or lordotic deformity in the thoracic, thoracolumbar or lumbar in the whole spine radiographs; (4) a history of spinal trauma and any operations for trauma, infection, or tumour; and (5) any symptom related to the spine according to their electronic medical records. A total of 335 subjects (182 males and 153 females) were finally included in this study. To compare the values of the occipital parameters with those in Kim HJ *et al*.’s study^10^, all of the subjects were divided into three age groups (group A: < 40, group B: 40~60, group C: > 60). Informed consent was obtained from all subjects.

### Radiographic assessment

**Figure 1:**
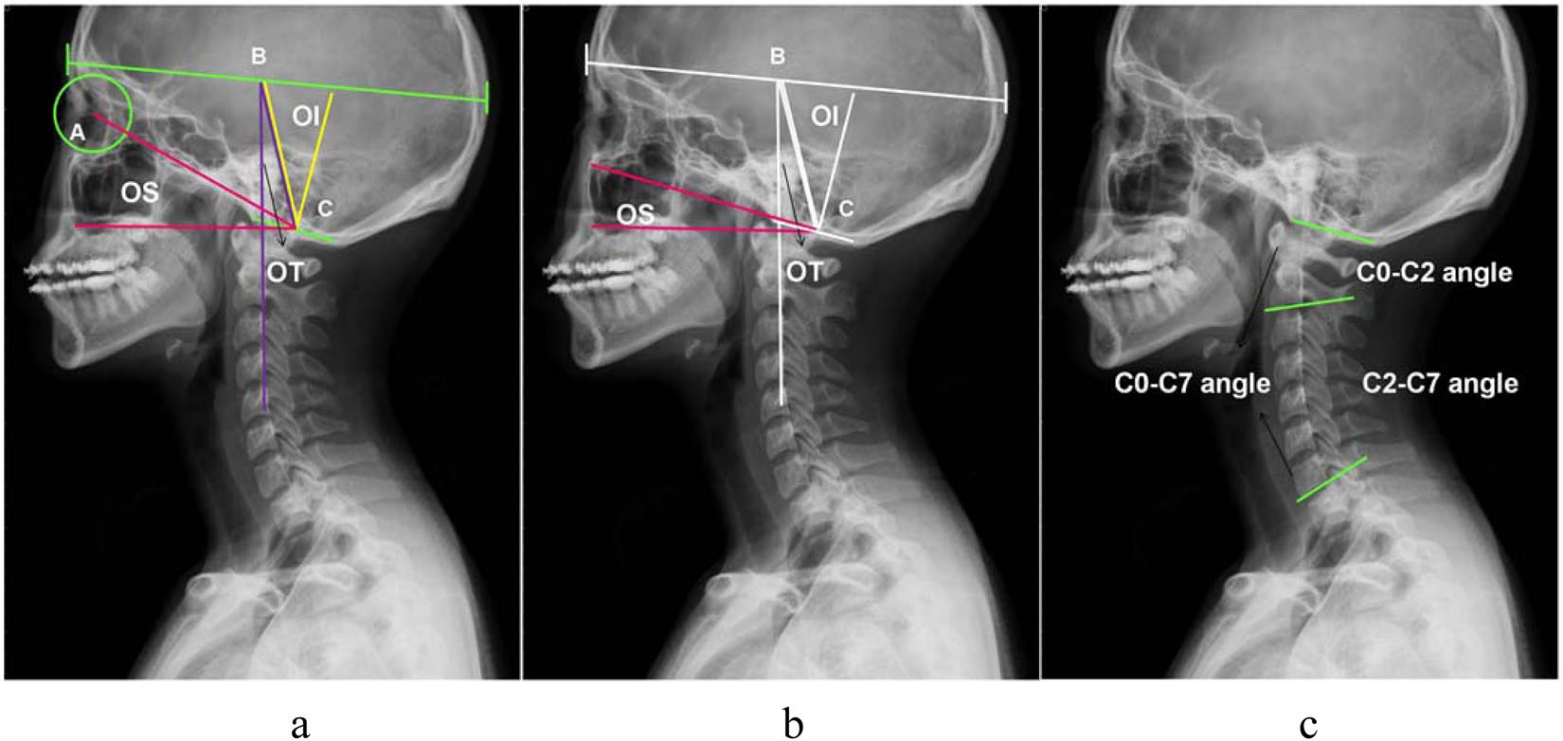
Measurement of the occipitocervical parameters. (**a**) A: Center of Orbit, B: Center of Skull, C: Center of Foramen Magnum. OI = angle subtended by BC and the perpendicular to foramen magnum (clivus to opisthion) [morphometric]. OS = angle between AC and a horizontal axis [non-morphometric]. OT = angle between BC and a vertical line[non-morphometric]. (**b**) New definition of OS. OS = angle between foramen magnum and a horizontal line [non-morphometric]. (**c**) C0-C2 angle = angle between the plate of foramen magnum and the inferior endplate of C2, C2-C7 angle = angle between the inferior aspects of vertebral bodies of C2 and C7 and C0-C7 angle = angle between the plate of foramen magnum and the inferior end plate of C2.

According to Kim *et al*.’s^10^ definition, OI was defined as the angle subtended by a line from the centre of the skull to the centre of the foramen magnum and perpendicular to the foramen magnum. The centre of the skull was defined as the middle between the anterior margin of the frontal sinus and posterior margin of the occiput. OS was defined as the angle between a line from the centre of the orbit to the centre of the foramen magnum and a horizontal axis. OT was defined as the angle between a line from the centre of the skull to the centre of the foramen magnum and a vertical line that originated from the centre of the skull (Fig. 1a). However, there was no geometrical equality relationship in these occipital parameters like in the pelvic parameters. We redefined OS as the angle between the foramen magnum and a horizontal line, which made the radiographic measurement more reproducible (Fig. 1b). According to our new definition, the geometric construction by complementary angles revealed that OI was the algebraic sum of OS and OT: Occipital incidence = occipital slope + occipital tilt.

As an orientation parameter, CL was divided into upper CL (C0–C2 angle) and lower CL (C2-C7 angle)^9^. The C0–C2 angle was formed by the plate of the foramen magnum and inferior endplate of C2. The C2-C7 angle was formed by the inferior aspects of the vertebral bodies of C2 and C7. The C0–C7 angle was the angle between the plate of the foramen magnum and inferior endplate of C2^9^ (Fig. 1c). In addition, T1S denoted the angle formed between the horizontal plane and inferior endplate of T1. TK was the angle between the superior endplate of T5 and inferior endplate of T12. A lordotic angle was assigned a positive value, and a kyphotic angle was assigned a negative value.

**Figure 2:**
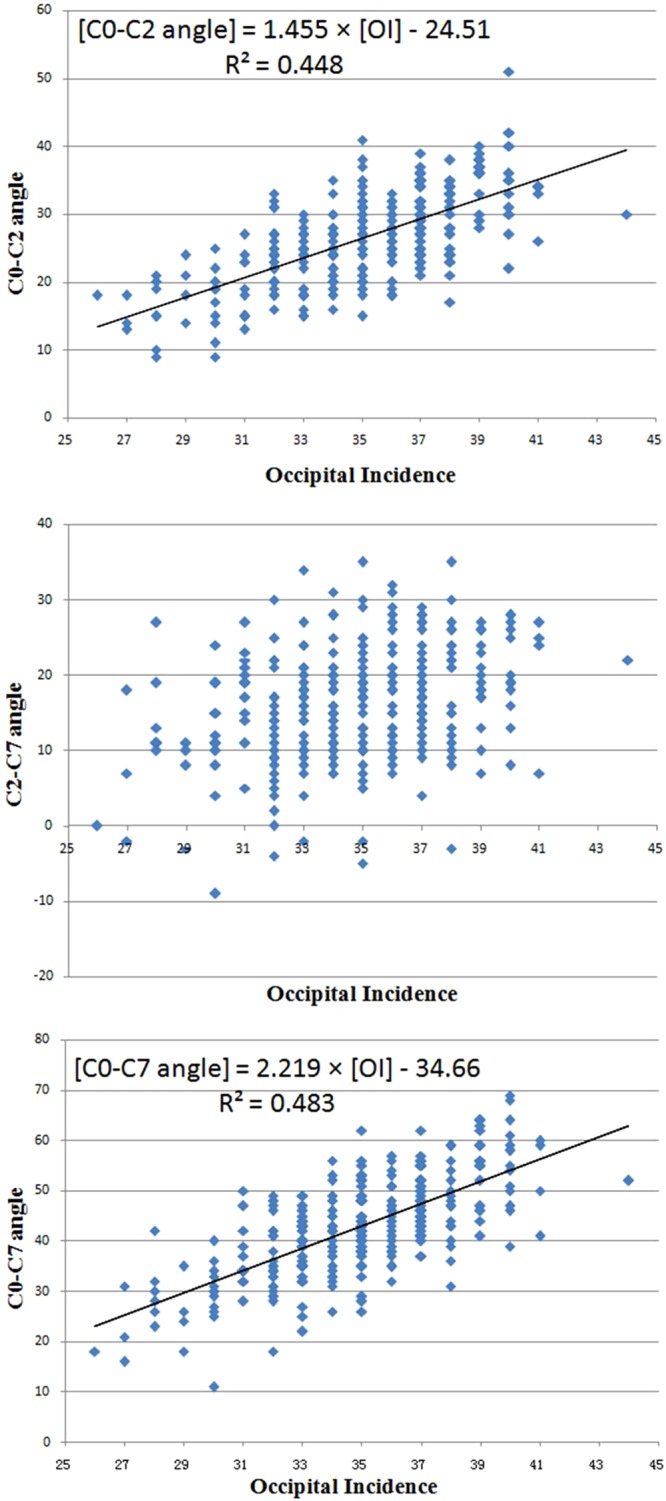
The scatterplots of OI and cervical parameters. (**a**) and (**c**) showed two significant linear shapes between two pairs of parameters (OI vs C0-C2 angle, OI vs C0-C7 angle), which represented the linear correlations. However, no linear correlation was showed between OI and C2-C7 angle (**b**).

Cervical parameters were plotted against OI for each subject. Then a linear trend line to the scatterplots was fitted by least-squares regression with an algorithm resident in the Excel spreadsheet (Microsoft, Redmond, Washington) (Fig. 2a,c). The formula of the linear trend line represented the linear regression model of cervical parameters and OI.

Two spine surgeons (Y. L. and W. Z.) independently and blindly performed the measurements at the same time to analyze the inter-observer reliability. In addition, these radiographic parameters were measured repeatedly by Y. L. after four weeks to analyze the intra-observer reliability with his twice measurements.

### Statistical analysis

All values were verified to be normal distributed by the one-Sample K-S test and are expressed as the mean ± standard deviation. The intra- and inter-observer reliabilities of the parameters were analysed using intraclass correlations. The correlations among the parameters were analysed with the Pearson correlation coefficient. A comparison of the parameters among three age groups (<40, 40~60, >60 years) was performed with one-way ANOVA, and differences among these parameters between females and males were analysed with Student’s t test. All statistical analyses were performed with SPSS version 19.0 (SPSS Inc, Chicago, IL), and a P-value < 0.05 was considered significant.

## Result

### Occipitocervical and thoracic parameters

Substantial intra- and interobserver reliability was found for the occipitocervical and thoracic parameters (Table 1), which showed that the redefined occipital and other radiographic measurement had good reproducibility.

**Table 1:** The intraclass and interclass correlation coefficients of radiographic measurements.

Parameters	Intra-observer reliability		Inter-observer reliability	
	Intraclass Correlation	95% Confidence Interval	Intraclass Correlation	95% Confidence Interval
OI	0.814	0.516–0.928	0.715	0.461–0.890
OS	0.806	0.614–0.912	0.742	0.505–0.901
OT	0.886	0.735–0.926	0.816	0.656–0.902
C0-C2	0.856	0.725–0.921	0.836	0.751–0.914
C2-C7	0.843	0.637–0.909	0.894	0.754–0.927
C0-C7	0.827	0.618–0.891	0.858	0.737–0.886
T1S	0.841	0.713–0.916	0.855	0.721–0.913
TK	0.890	0.741–0.930	0.881	0.750–0.922

Abbreviation: Occipital incidence (OI), occipital slope (OS), occipital tilt (OT), C0-C2 angle (C0-C2), C2-C7 angle (C2-C7), C0-C7 angle (C0-C7), T1 slope (T1S), thoracic kyphosis (TK).

OI, OS, and OT in the 335 normal subjects averaged 34.6° (range 26°–40°), 23.4° (range 14°–55°), and 11.8° (range 2°–22°), respectively (Table 2). OI was not influenced by age, with no significant difference among groups A, B and C (Table 3, P > 0.05). Compared with group A, however, OS and OT were found to be larger and smaller in the two senior groups, respectively (Table 3, P < 0.05). Further, no gender differences were noted in OI, OS or OT for the whole cohort (P > 0.05, Table 4).

**Table 2:** Occipitocervical and Thoracic Parameters.

	Age (year)	OI (°)	OS (°)	OT (°)	C0-C2 (°)	C2-C7 (°)	C0-C7 (°)	T1S (°)	TK (°)
Mean	41.6	34.6	23.4	11.8	25.8	15.6	41.4	28.7	31.7
SD	12.3	3.1	7.4	3.3	8.8	10.5	10.1	7.3	10.9
range	18–75	26–44	14–55	2–22	10–51	−17–45	25–69	8–50	12–59

Abbreviation: Refer to Table 1.

**Table 3:** Occipitocervical and Thoracic Parameters between Three Age Groups.

Parameters	Mean ± SD (°)			*p*
	<40 (86)	40~60 (133)	å 60 (116)	
OI	34.4 ± 3.2	35.2 ± 3.2	34.6 ± 2.7	P = 0.441
OS	20.7 ± 8.8	25.7 ± 7.3	24.8 ± 6.4	P = 0.032*
OT	13.2 ± 4.5	9.0 ± 2.7	9.3 ± 3.0	P = 0.027*
C0-C2	25.9 ± 9.4	27.0 ± 8.5	24.6 ± 9.6	P = 0.195
C2-C7	12.7 ± 11.2	16.0 ± 10.7	21.6 ± 9.6	P <0.001*
C0-C7	37.6 ± 10.4	43.0 ± 10.5	45.2 ± 9.6	P = 0.008*
T1S	23.7 ± 5.3	26.5 ± 8.0	30.1 ± 7.3	P = 0.025*
TK	22.4 ± 9.2	29.4 ± 9.2	43.9 ± 12.2	P < 0.001*

Abbreviation: Refer to Table 1.

**Table 4:** Occipitocervical and Thoracic Parameters between Female and Male.

Parameters	Mean ± SD (°)		*p* Value
	Female(182)	Male (153)	
OI	34.3 ± 2.9	34.9 ± 3.3	0.601
OS	23.1 ± 7.7	22.7 ± 7.0	0.184
OT	10.8 ± 3.0	12.3 ± 4.2	0.373
C0-C2	27.4 ± 8.7	24.1 ± 8.7	0.007*
C2-C7	13.9 ± 11.6	18.1 ± 9.8	0.007*
C0-C7	41.3 ± 11.3	42.2 ± 9.7	0.577
T1S	29.2 ± 7.1	27.9 ± 7.4	0.448
TK	33.6 ± 13.2	30.6 ± 9.8	0.115

Abbreviation: Refer to Table 1.

The mean C0–C2 angle was 25.8° (range 10° to 51°), the mean C2-C7 angle was 15.6° (range −17° to 45°), and the mean C0-C7 angle was 41.4° (range 25° to 69°) for the whole cohort (Table 2). The C2-C7 angle and the C0-C7 angle were both found to be significantly larger with age, whereas the C0-C2 angle did not show any difference among the three age groups. In addition, a larger C0-C2 angle and smaller C2-C7 angle were observed in females compared with males (P < 0.05), while no gender difference was noted in the C0-C7 angle (Table 4).

The average T1S and TK values were 28.7° (range 8° to 50°) and 31.7° (range 12° to 59°), respectively (Table 2). T1S and TK showed a significant increase with age (P < 0.05, Table 3). No gender differences were observed in these two thoracic parameters (P > 0.05, Table 4).

### Correlations of the Parameters

As showed in Table 5, OI was significantly correlated with OS (r = 0.279), the C0-C2 angle (r = 0.573), the C2-C7 angle (r = 0.240) and the C0-C7 angle (r = 0.589). OS was also significantly correlated with OT (r = 0.885), the C0-C2 angle (r = 0.327), the C2-C7 angle (r = 0.300) and the C0-C7angle(r = 0.478). Meanwhile, correlations were also found between OT and the C0-C2 angle (r = 0.172), the C2-C7 angle (r = 0.230) and the C0-C7 angle (r = 0.313). With regression analyses, statistically significant linear regression models were established as follows: [C0-C2 angle] = 1.455 × [OI] −24.51, [C0-C7 angle] = 2.219 × [OI] −34.66 (Fig. 2).

**Table 5:** The Pearson Correlation Coefficient and P-value.

Parameters		Age	OS	OT	C0-C2	C2-C7	C0-C7
OI	r p	−0.045 0.430	0.279 0.000*	−0.051 0.826	0.573 0.000*	0.240 0.000*	0.589 0.000*
OS	r p	0.137 0.016*		0.885 0.000*	0.327 0.000*	0.300 0.000*	0.478 0.000*
OT	r p	0.160 0.005*			0.172 0.001*	0.230 0.000*	0.313 0.000*
T1S	r p	0.125 0.028*	0.092 0.176	0.059 0.634	0.101 0.106	0.484 0.000*	0.355 0.000*
TK	r p	0.232 0.000*	0.077 0.435	0.072 0.508	0.080 0.341	0.331 0.000*	0.289 0.000*

Abbreviation: Refer to Table 1.

Furthermore, both T1S and TK were found to be correlated with the C2-C7 angle (r = 0.484 and 0.331, respectively) and C0-C7 angle (r = 0.355 and 0.289, respectively). However, no significant correlation was noted between the thoracic parameters and the C0-C2 angle and the occipital parameters.

## Discussion

Over the last decade, multiple publications have explored the sagittal profile of the thoracolumbar spine^13–15^. Compared to the thoracic and lumbar spine, the guidelines for the assessment of the sagittal alignment of the cervical spine have not been clearly defined. In 1995, Hilibrand *et al*.^16^ reported a phenomenon that a hypo-kyphotic thoracic spine accompanies a kyphotic cervical spine. With the establishment of spinal and pelvic sagittal radiographic parameters^11,17^, a correlation between the cervical sagittal curvature and thoracolumbar and pelvis had been gradually demonstrated^18–20^. However, the cervical spine is a remarkably complex segment as many factors influence its alignment and balance. Recently, Lee *et al*.^8,9^ investigated the craniocervical sagittal alignment in 77 asymptomatic adults, in which cranial offset, cranial tilting and cervical tilting were found to be significantly correlated with the cervical sagittal curvature. Le Huec *et al*.’s^21^ study demonstrated a close correlation between the C7 slope and cranio-cervical system with cranial incidence and the spino-cranial angle. At present, the compensatory mechanism of the cervical sagittal alignment is still unclear. In addition, the influence of the occipital orientation on the sagittal curvature of the cervical spine has not been explored.

Aiming to establish an anatomic parameter in the occiput, like PI for cervical, Kim *et al*.^10^ proposed the occipital incidence, which is considered to be intimately related to the cervical spine alignment and global sagittal balance. In their study,  OI was not affected by age (OI = 34.3 ± 4.3, 35.1 ± 3.9, 35.1 ± 3.6 in age groups < 40, 40–60 and >60, respectively), OS was decreased and OT was increased in all age groups (OS = 22.6 ± 8.0, 24.3 ± 7.9, 28.2 ± 7.5 and OT = 12.2 ± 6.9, 10.8 ± 7.7, 5.9 ± 8.1 in age groups < 40, 40–60 and >60, respectively). In order to make OI was the algebraic sum of OS and OT like the relationship between the pelvic parameters, we redefined OS as the angle between the foramen magnum and a horizontal line. Our results showed that the values of OI and OT in the current study were similar to their values (Table 3). The value of the newly defined OS was theoretically smaller than that of the old definition of OS. Consistently, OI was also constant when age increased (P > 0.05). However, OS was larger and OT was smaller in senior groups compared with younger group (P < 0.05, Table 3). Meanwhile, the OI, OS and OT values in this study were not influenced by gender, which was not evaluated in Kim *et al*.’s study^10^ (P > 0.05, Table 3).

**Figure 3:**
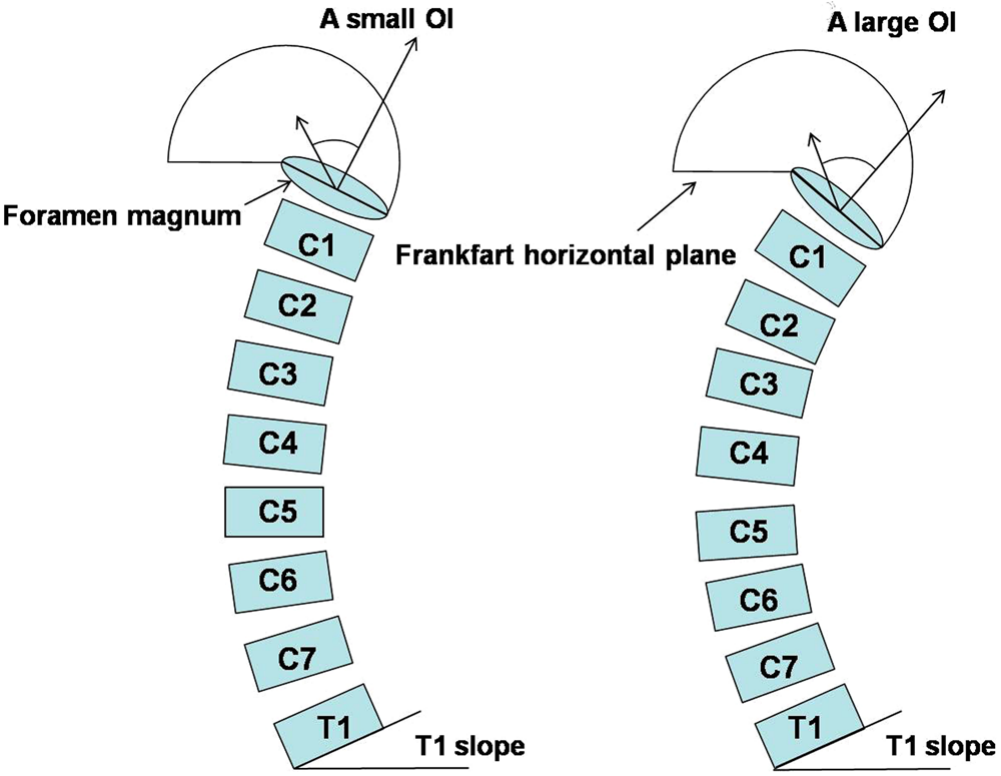
A illustration of the relationship between occipitocervical alignment and CL. When T1 slope remained unchanged, a large OI (meant that the foramen magnum was more slope) would increase CL in order to balance the head and maintain a horizontal gaze.

The values of the regression coefficients emphasize the close relationship between the occipital parameters and cervical curves. Statistically, analysis of the Pearson correlation coefficient in this study showed that OI was strongly correlated with the C0-C2 angle (r = 0.573), C2-C7 angle (r = 0.240) and C0-C7 angle (r = 0.589). In this regard, any change in one of these parameters induced a change in the others, except for the OI, which was the only anatomical and constant parameter, while the others were positional parameters. However, compared with the C0-C2 angle and C0-C7 angle, the correlation coefficient between the C2-C7 angle and OI was weaker. According to previous results^18–20^, we determined that the influence of the sub-axial spine on the C2-C7 angle weakened its correlation with OI. Based on our results, we presumed that the occipital orientation could balance the sagittal alignment of the cervical spine with minimum energy expenditure as compensatory changes to maintain the head over the pelvis and maintain a horizontal gaze. Compared to a small OI alignment, a large OI yields a steep slope in the foramen magnum and a large cervical curve, with the T1 slope remaining unchanged, and vice versa (Fig. 3). Further, the scatterplots of OI and the cervical parameters showed two significant linear shapes between the two pairs of parameters (OI *vs* the C0-C2 angle, OI vs the C0-C7 angle). OI played a major role in the degree of lordotic curvature of the cervical spine, similar to that of PI and lumbar lordosis. From regression analysis, statistically significant linear regression models were postulated as follows: [C0-C2 angle] = 1.455 × [OI] −24.51 and [C0-C7 angle] = 2.219 × [OI] −34.66 (Fig. 2). Through the two predictive equations, a physiological alignment of the cervical spine was predicted by OI, which may serve as baseline data for the evaluation of cervical sagittal curves. Such relationships between the occipital orientation and cervical sagittal alignment should be taken into consideration when performing cervical fusion, especially in occipitocervical fusion surgery.

Moreover, the next important point discussed in our study was the compensatory mechanism of the upper and lower cervical sagittal alignment. As reported in previous studies, the upper cervical sagittal alignment is not correlated with the sub-axial spine or pelvic alignment. Abelin-Genevois *et al*.^22^ studied the cervical spine alignment in 150 asymptomatic patients and found no direct correlation between the upper cervical sagittal angles and thoracolumbopelvic parameters. Lee *et al*.’s study^8,9^ also demonstrated that the C0-C2 angle did not correlate with the thoracolumbar or pelvic profile. Similarly, the C0-C2 angle in the present study did not show any correlation with the thoracic alignment (Table 5). However, our results revealed that the upper cervical sagittal alignment was influenced by the occipital sagittal orientation: OI (r = 0.573), OS (r = 0.327) and OT (r = 0.172) had significantly positive correlations with the C0-C2 angle (Table 5), meaning that the C0-C2 angle increased with increased OI, OS or OT. The influence of the thoracic alignment on the lower cervical sagittal alignment (C2-C7 angle) is well known, which was demonstrated in this study (Table 5) and in previous reports^8,9,11,16,17^. In addition, our work further revealed the correlation of the occipital orientation with the C2-C7 angle. In this regard, compared to the upper cervical spine, the compensatory mechanism of the sagittal alignment in the lower cervical was more complex, which helps to understand that there was no linear correlation between OI and the C2-C7 angle (Fig. 2). Our results reminded us that the upper cervical sagittal alignment was mainly compensated by the occipital orientation instead of the thoracic alignment, while the lower cervical sagittal alignment was mainly compensated by the above two factors.

The cervical spine is complex, and surgical management of cervical disease remains a significant challenge. An understanding of cervical biomechanics as well as the normative data for cervical alignment is necessary to manage complex cervical pathology. To the best of our knowledge, this is the first study to systematically investigate the effect of the occipital orientation on the cervical spine. The results of our study demonstrated that the occipital orientation was significantly correlated with the cervical sagittal curvature. Accordingly, there was also a linear correlation between OI and cervical lordosis like that between PI and lumbar lordosis, which could serve as a guideline for the assessment of the sagittal alignment of the cervical spine and the reconstruction of lordosis during surgery. Furthermore, it is very important to remember that the spinal regions are not independent of one another. Cervical lordosis not only depends on both thoracic kyphosis and lumbar lordosis but also has a relationship with the occipital orientation. Cervical lordosis can be considered to be an adaptation by which the cervical spinal segment changes relative to the occipital orientation and the other spinal segments to attempt to maintain the head over the pelvis and maintain a horizontal gaze.

There were two limitations to our study. First, a group of healthy volunteers would be ideal, but this criterion would be unethical for radiation protection reasons. Second, a longitudinal study is preferable to clarify the relationship between age and the variations in occipital parameters. Three, there is a possibility that the occipital orientation differs between different races, which need to be further studied.

## Conclusions

OI is an anatomic morphology parameter with no differences between ages and genders. This study demonstrates the key importance of the occipital orientation in the evaluation of the cervical sagittal alignment. A more oblique occipital orientation causes the cervical spine to become more lordotic to maintain a forward gaze, with an unchanged T1 slope. The upper cervical sagittal alignment is mainly compensated by the occipital orientation, while a lower cervical sagittal alignment is mainly compensated by the occipital and thoracic alignments. These results could help to better understand the cervical sagittal profile and serve as baseline data for planning an optimum sagittal fusion angle in the cervical spine.
